# Radiation Dose-Dependent and -Independent Pulmonary Infiltrates in Patients with High-Grade Pneumonitis After Radiochemotherapy and Durvalumab Consolidation for Stage III NSCLC

**DOI:** 10.3390/diagnostics16060827

**Published:** 2026-03-11

**Authors:** Andreas Herz, Aymane Khouya, Maja Guberina, Martin Metzenmacher, Marcel Opitz, Christoph Pöttgen, Gerrit Fischedick, Hubertus Hautzel, Thomas Gauler, Ken Herrmann, Erik Büscher, Servet Bölükbas, Fabian Doerr, Natalie Baldes, Laura Valentina Klüner, Benedikt M. Schaarschmidt, Rüdiger Karpf-Wissel, Jane Winantea, Denise Bos, Verena Jendrossek, Emil Mladenov, Lena Gockeln, Mario Andre Hetzel, Florian Wirsdörfer, Martin Schuler, Martin Stuschke, Nika Guberina

**Affiliations:** 1 Department of Radiotherapy, West German Cancer Center, University Hospital Essen, University Duisburg-Essen, 45147 Essen, Germany; andreas.herz@uk-essen.de (A.H.); christoph.poettgen@uk-essen.de (C.P.);; 2 National Center for Tumor Diseases (NCT), NCT West, West German Cancer Center, 45147 Essen, Germany; 3 German Cancer Consortium (DKTK), Partner Site University Hospital Essen, 45147 Essen, Germany; 4 Department of Medical Oncology, West German Cancer Center, University Hospital Essen, 45147 Essen, Germany; martin.metzenmacher@uk-essen.de; 5 Department of Diagnostic and Interventional Radiology and Neuroradiology, University Hospital Essen, 45147 Essen, Germany; 6 Department of Nuclear Medicine, University Hospital Essen, 45147 Essen, Germany; 7 Department of Pulmonary Medicine, Section of Interventional Pneumology, West German Cancer Center, University Medicine Essen-Ruhrlandklinik, University Duisburg-Essen, 45147 Essen, Germany; erik.buescher@rlk.uk-essen.de (E.B.);; 8 Department of Thoracic Surgery and Thoracic Endoscopy, West German Cancer Center, University Medicine Essen-Ruhrlandklinik, University Duisburg-Essen, 45147 Essen, Germany; 9 Institute of Cell Biology (Cancer Research), University Hospital Essen, 45147 Essen, Germany

**Keywords:** pneumonitis, lung cancer, radiation therapy, immunotherapy, durvalumab consolidation

## Abstract

**Background/Objectives**: Analysis of the density and spatial distribution of pulmonary infiltrates of patients with high-grade (≥3) pneumonitis after radiochemotherapy and durvalumab consolidation (RT/CTx + IO) was performed in order to define dosimetric hallmarks of the development of infiltrates following this multimodality treatment. **Methods**: Consecutive patients treated with RT/CTx + IO for stage III NSCLC were retrospectively reviewed with respect to the occurrence of grade ≥ 3 pneumonitis. Lung infiltrates were contoured on follow-up CT scans acquired around the time of maximum pneumonitis expression. The applied dose distribution was overlaid with the follow-up CT using elastic deformation, and infiltrates were binned according to their density in density strata of 50 HU. The dose and density dependence of partial infiltrate volumes per unit lung volume was analyzed using a mixed fixed and random effect model adjusting for patient, density and dose-dependent random effects. **Results**: Six patients with grade ≥ 3 pneumonitis were identified from 132 patients treated with RT/CT + IO at a comprehensive cancer center. Partial volumes of lung infiltrates captured by follow-up CT with maximum pneumonitis expression ranged from 15.5 to 60.0% (median 39.8%). A significant, systematic dose–response relationship was found for partial lung infiltrate volumes per dose and density bin. A unimodal density distribution of partial lung infiltrate volumes was also found over the infiltrate density range of −1000 to 100 HU. This was determined using a mixed model that adjusted for random effects (*p* < 0.0001 for both effects, F-test). There was no interaction effect between systematic dose and infiltrate density dependence of the partial infiltrate volumes. The proportion of infiltrate volumes that are attributable to the systematic dose–response relation amounts to a mean of 16.6% of the total infiltrate volume per patient according to this model. Compared to patients with pneumonitis of grade ≤ 2, patients with high-risk pneumonitis had higher partial infiltrate volumes, particularly in the low-dose regions in five grade dose bins up to 20 Gy (AUC = 1.0, *p* < 0.0001, likelihood-ratio test). **Conclusions**: Dose-dependent and -independent partial lung infiltrate volumes were found in patients with high-grade pneumonitis after RT/CTx + IO. These results indicate that pneumonitis involves contributions from both radiochemotherapy-induced and immunotherapy-related mechanisms.

## 1. Introduction

Concurrent radiotherapy and chemotherapy followed by consolidation therapy with PD-L1 antibodies have become the preferred standard treatment for patients with locally advanced non-small cell lung cancer (NSCLC) with a PD-L1 tumor proportion score > 1% and without targetable EGFR mutation or ALK alterations [1]. Several me-ta-analyses found pooled mean grade ≥ 3 pneumonitis incidences between 5.7% and 8% [2,3,4,5]. After such a combined therapy schedule, radiation-induced pneumonitis was defined in some studies as infiltrates within radiation fields and checkpoint inhibitor-based pneumonitis as infiltrates not specifically located within the radiation fields [6]. However, pulmonary infiltrates can sometimes also occur in the low-dose region in the lung or outside the radiation fields [7,8,9,10]. Like radiation therapy-induced changes, CT findings described in patients with immune checkpoint-related pneumonitis cover a whole range of different patterns [11,12]. Our study addresses this difficulty in clinical routine by characterizing CT patterns associated with high-grade pneumonitis in patients treated with induction chemotherapy, definitive radio(chemo-)therapy, and subsequent immunotherapy consolidation for locally advanced NSCLC.

Wang et al. systematically reviewed publications aimed at predicting radiation- or checkpoint inhibitor-induced pneumonitis by clinical, radiomic and dosiomic variables using machine learning methods [13]. Several studies on the prediction of check-point-inhibitor-induced pneumonitis used information from pretreatment CT scans in addition to clinical data for prediction of pneumonitis [14,15,16,17,18,19]. Radiomic and deep learning features could improve the prediction of checkpoint-induced pneumonitis over clinical and laboratory parameters alone, achieving precision characterized by pooled areas under the curve (AUC) of 0.86. Some deep learning models for radiation pneumonitis also included follow-up CT scans for training the classifier [20]. Studies including dosiomic or radiomic data from the planning CT to predict radiation pneumonitis achieved median precisions characterized by AUCs between 0.76 and 0.83 [21]. While individual fixed dosimetric parameters have limited predictive value for pneumonitis prediction [22,23,24,25,26], prediction can be improved using additional radiomic parameters [13,21].

Previous studies primarily focused on pretreatment imaging, including CT and molecular imaging [27]. Several studies analyzed lung density changes on follow-up CT scans compared to the pretreatment CT scans in dependence on the radiation dose that a lung voxel received after radio- or combined radiochemotherapy [28,29,30,31,32]. Here, a monotonously increasing dose–response relation after radiotherapy without immu-notherapy was detected that was also dependent on the follow-up time [28,29,30,31,32]. In contrast, our study examines post-treatment CT patterns of radiation- and immuno-therapy-induced pneumonitis. While some post-treatment imaging studies exist in patients receiving immuno(chemo-)therapy without radiotherapy [33], data in the combined radio(chemo-)immunotherapy setting remain scarce [34,35,36]. In this study, we investigate the presence of an intra-patient radiation dose–response relationship for the partial volume of pulmonary infiltrates in patients who developed a high-grade (Grade ≥ 3) pneumonitis after induction chemotherapy, concurrent radiochemotherapy and durvalumab consolidation therapy according to the Pacific trial for locally ad-vanced NSCLC. A systematic dose–response relation is considered strong evidence for causality between an agent and its considered effect [37,38,39]. We hypothesize that the variance components of the random effects are not zero. From the proportion of infiltrates that systematically follow a radiation dose–response, we aimed to characterize the contribution of radiotherapy to high-grade pneumonitis after combined radiochemotherapy and consolidation immunotherapy and to describe the hallmarks of this type of pneumonitis after combined treatment.

## 2. Materials and Methods

### 2.1. Participants and Radiation Therapy

All treated patients gave their consent to the treatment and took part in the prospective, institutional clinical registry trial (18-8364-BO). In this retrospective analysis approved by the institutional ethics committee (23-11186-BO), a total of 132 consecutive patients with inoperable stage III NSCLC treated with definitive radiochemotherapy and durvalumab consolidation at a comprehensive cancer center between 2018 and 2023 were included. Patients had to fulfill the inclusion and exclusion criteria of the Pacific trial and did not have a clinical diagnosis of interstitial lung disease [1]. All patients received three cycles of induction chemotherapy, containing the doublet cisplatin/taxol, and concurrent radiochemotherapy with cisplatin/vinorelbine [40]. Cisplatin was substituted by carboplatin in patients with impaired kidney function. Patients were treated with image-guided radiotherapy (IGRT), and either volumetric arc therapy (VMAT) or multifield intensity modulated radiotherapy (IMRT) to achieve a conformal dose distribution, ensuring sparing of vulnerable structures including lung parenchyma. Respiratory-gated radiotherapy was performed in inspiratory deep breath hold or in prospective exhalation. Online navigation was conducted using a 6-degree-of-freedom table on a TrueBeam linear accelerator (Varian, CA, USA). Dose distributions were calculated using an analytical anisotropic algorithm with heterogeneity correction (AAA). After radiochemotherapy completion, patients underwent durvalumab consolidation. The immunotherapy dosage was 10 mg/kg body weight administered every 2 weeks or a fixed dose of 1500 mg administered every 4 weeks. Treatment was continued until disease progression, the occurrence of unacceptable toxicity, or for a maximum duration of 12 months. In clinically stable patients, it was recommended that therapy be continued at the first signs of disease progression until progression was confirmed. At the start of treatment, monitoring was performed on a weekly basis, later once every two weeks.

### 2.2. Study Design

Pulmonary infiltrates of patients with high-grade pneumonitis receiving definitive radiochemotherapy and durvalumab consolidation were characterized with respect to their density distribution and dose–response relation. Grading of clinical pneumonitis was performed according to the Common Terminology Criteria for Adverse Events (CTCAE) version 5.0. For this, medical records were analyzed in detail with a focus on maximum symptoms over time, diagnostic investigations, and applied treatment strategies to verify the pneumonitis diagnosis. A pneumonitis of grade 3–5 was classified as high-grade pneumonitis. Pulmonary infiltrates were contoured in the follow-up CT at the time point of maximum expression by consensus among two expert chest radiologists and a radiation oncologist. Delineation of infiltrates was supervised manually using semi-automated functions such as image thresholding using predefined density thresholds as described previously [41]. All follow-up CT studies of the patients with high-grade pneumonitis were performed with i.v. contrast as CT pulmonary angiograms.

All radiologic features of pulmonary infiltrates, characteristic of non-fibrotic and fibrotic pneumonitis, were delineated. These included four patterns: patchy ground-glass opacities, patchy consolidation and ground-glass opacities, diffuse ground-glass opacities and diffuse consolidations [42].

### 2.3. Infiltrate Separation into Density Strata and Generation of Dose-Volume Histograms

All CT images at the time of maximum pulmonary infiltrate expression captured by a CT scan (referred to as infiltrates-CT) were identified from the medical history by two expert radiologists and radiation oncologists. The respective images were exported in DICOM format to the MIM Maestro software (version 7.3.2, MIM Software Inc., Cleveland, OH, USA). Lung infiltrates were contoured by the two observers by consensus, both with more than 10 years of experience. The original planning CT (pCT), which was used for calculation of the dose distribution and treatment planning, was deformed onto the infiltrates-CT using a hybrid deformable image registration (DIR) approach in MIM Maestro, relying on density information and contoured lung structures [43]. For the hybrid deformable image registration approach, the lung surface was manually corrected following AI-based contouring using the Spyglass approach in MIM. Anatomical landmarks, including large vessels, bronchial bifurcations, and interlobar fissures, were identified, and the resulting deformation was interactively re-viewed and, when necessary, corrected by a radiation oncologist. This process was performed using the user-guided deformation re-finement tool, as previously described in [44,45]. The 3D dose distribution and the contoured structures were then transferred from the pCT to the infiltrates-CT using the obtained deformation matrix. The analyzed structures included the abnormal lung parenchyma excluding the planning target volume (PTV), referred to as INF_PTV, and a complementary structure consisting of the lung volume minus both the PTV and the infiltrates (Lungs_PTV_INF). In order to facilitate voxel and HU—based analyses, the infiltrates-CT scans were down-sampled to an isotropic grid of 128 × 128 × 66 voxel size in the Dicom xyz-coordinate system in MATLAB (version R2020b; MathWorks Inc., Natick, MA, USA). The resulting voxel sizes then ranged from 3.2 mm × 3.3 mm × 4.3 mm to 3.9 mm × 3.9 mm × 5.1 mm from patient to patient. The corresponding down-sampled 3D coordinates of the dose grid were resampled into 0.1 Gy intervals using the DICOM DoseGridScaling factor. Each voxel was characterized by the spatial xyz-coordinates (X, Y, Z), the HU and the physical dose values. A separate differential dose-volume histogram was generated for the INF_PTV and Lungs_PTV_INF contours over each 50 HU density stratum. The INF_PTV and Lungs_PTV_INF contours’ coordinates from the DICOM RTSTRUCT linked to the original infiltrates-CT were processed using MATLAB’s in polygon function and adapted to the down-sampled CT grid. A voxel in the down-sampled grid was assigned to the infiltrate structure if more than 50% of its volume was covered by the original contour. This was achieved by applying nearest-neighbor interpolation to the binary mask derived from the original structure contours. Infiltrate volumes were subdivided according to their density into 50 HU steps in the range from −1000 to +100 HU. Pulmonary arteries with higher densities were therefore excluded from the infiltrates.

### 2.4. Statistical Analysis

All statistical analyses were performed using the SAS statistical software system (SAS/STAT 15.1, SAS Institute Inc., Cary, NC, USA). The primary endpoint of the study was the analysis of the existence of a dose–response relationship. The independence from dose should be rejected at alpha = 0.01. If a dose dependence of the percentage infiltrates per dose and HU bin could be demonstrated, sequential testing was allowed, using alpha spending. Otherwise, the analysis stopped. AUC values were considered significant at alpha = 0.002 to account for multiple testing according to the conservative Bonferroni correction. For comparison of different fits, the Akaike Information Criterion (AIC) was used. The dose-volume histograms from the INF_PTV and Lungs_PTV_INF volumes of patients with high-grade pneumonitis were simultaneously analyzed with a linear mixed fixed and random effect model over all HU density strata (Procedure Mixed from SAS). The endpoint of this study is defined as the ratio of the volume of infiltrates with a given density within a specified dose bin (INF_PTV_HU_dosebin_) to the total lung volume outside the PTV that received the same binned dose (INF_PTV_dosebin_ + Lungs_PTV_INF_dosebin_). These volume ratios are termed partial volumes of infiltrate per dose and HU bin. The number of Hounsfield unit (HU) bins was determined such that the interquartile range of the density distributions was subdivided into at least five dose bins per patient, corresponding to a bin width of 50 HU. This way, density of the infiltrates was binned into different adjacent density bins of 50 HU from −1000 HU to 100 HU and used as a fixed effect classification variable in the model.

Dose steps from the dose-volume histograms had a step width of 0.1 Gy. To avoid presupposing a specific functional form of the dose–response relationship, a stepwise modeling approach using 5 Gy dose bins was employed. Thus, complexity and statistical fluctuations were controlled. Dose was binned into adjacent 5 Gy dose bins from >0 Gy to <5 Gy ([0–5) Gy) up to 50 Gy to <55 Gy and used as another fixed effect classification variable. This method provided a superior fit to the association between percentage of infiltrate and dose per bin compared with a linear–quadratic dose–response model in patients with grade 1–2 pneumonitis [41], as assessed using the Akaike Information Criterion (AIC). The number of degrees of freedom was constrained to minimize the risk of overfitting, thereby limiting the number of dose bins. Simultaneously, the dose binning needed to correspond to clinically relevant lung dose–volume metrics (V5, V20, and V25), for which established tolerance thresholds exist.

A random intercept model was used, using a HU-dependent random intercept and the crossed effect between patient and dose as a random subject. Weight factors were used for all partial volumes of infiltrates per dose and HU bin that were the Lung_PTV per dose and density bin, normalized by the total lung volume outside the PTV per patient. Hierarchical clustering was performed using the procedures Aceclus and Cluster from SAS. Ward’s minimum-variance clustering method was used. 2D and 3D plots were generated using the procedures G3d and Sgplot. Logistic regression and calculation of area under the curve values were performed with the procedure logistic.

## 3. Results

Between 2018 and 2023, 4.5% (6 out of 132) of this group of consecutive patients who received definitive radiochemotherapy and durvalumab consolidation according to the inclusion criteria of the Pacific trial at this institution developed a grade ≥ 3 pneumonitis. Table 1 summarizes the baseline characteristics and dosimetric parameters of these patients with grade ≥ 3 pneumonitis. Dosimetric parameters for lung exposure of these six patients with high-grade pneumonitis were not significantly different from those found in a recently reported series of 60 patients from this institution treated according to the Pacific regimen who had up to grade 2 pneumonitis (*p* = 0.1795 for mean lung dose, *p* = 0.3541 for the V20, *p* = 0.2273 for V5, Wilcoxon signed rank test). One further patient with initially suspected pneumonitis of grade ≥ 3 was excluded from this analysis after microbiological/virological proof of infectious pneumonia (herpes simplex virus (HSV) pneumonia), who responded to the respective therapy. As the diagnosis of a radiation- and immunotherapy-related pneumonitis requires a careful exclusion of any infectious focus, the following diagnostic investigations were performed: blood cultures (5 of 6 patients), virologic diagnostics (all patients), broncho-alveolar lavage and high-resolution chest CT scans.

The median time period between the beginning of symptoms and the CT scan with the maximum extension of infiltrates was 29 days (range: 2–56). All patients who developed a high-grade pneumonitis received oxygen and corticosteroid treatment. Broad-spectrum antibiotics were given to all patients. Three patients not responsive to corticosteroids also received mycophenolate mofetil (2 of 6 patients) or infliximab (1 patient). Two patients were intubated and received mechanical ventilation in an intensive care unit (1 patient for a total of 17 days until extubation and the second patient for 8 days until death due to an acute respiratory distress syndrome (ARDS)). Three patients were responsive to initiation of corticosteroid treatment and showed a good partial regression of clinical symptoms within several days, one patient showed prolonged dyspnoea and received professional in-hospital treatment. Two patients showed no residual symptoms and were not oxygen-dependent in the follow-up visits after determination of corticosteroid tapering. One patient received long-term oxygen therapy but presented no residual dyspnoea in the follow-up. One patient was discharged from the ward without oxygen-dependence but did not attend further follow-up visits. The median follow-up time from the last day of radiochemotherapy until the last follow-up CT scan was 402 days (range 46–1617 days).

**Figure 1:**
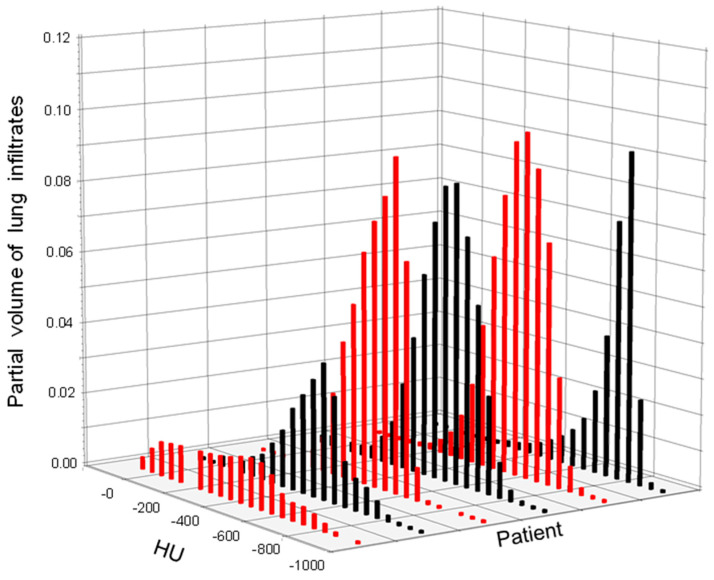
Density profile of partial lung volumes of infiltrates per patient. Differences in the distribution of the proportions of infiltrates over the HU between the patients were significant (*p* < 0.0001, chi2 test). Red and black color serve a better graphic clarity.

Figure 1 summarizes the partial volumes of lung infiltrates of indicated density over the 50 Hounsfield unit density bins at the time point of maximum infiltrate expression captured by a follow-up CT scan. The partial volumes of lung infiltrates in the total lung volume minus PTV (Lungs_PTV) ranged from 15.5% to 60.0% (median 39.8%) for the different patients with high-grade pneumonitis. There were significant differences in the location of the density distribution from patient to patient (*p* < 0.0001, row mean scores Cochrane–Mantel–Haenszel statistic). The predominant radiologic pattern of pneumonitis was organizing pneumonia (OP) in three patients with features of dystelectasis, parenchymal consolidation and ground-glass opacities (GGO). Two patients presented with acute interstitial pneumonitis (AIP); both developed tractions bronchiectasis (TBR) and extensive ground-glass opacities (GGO) following treatment. Another patient with nonspecific interstitial pneumonia (NSIP) showed a TBR pattern and GGO after therapy.

**Figure 2:**
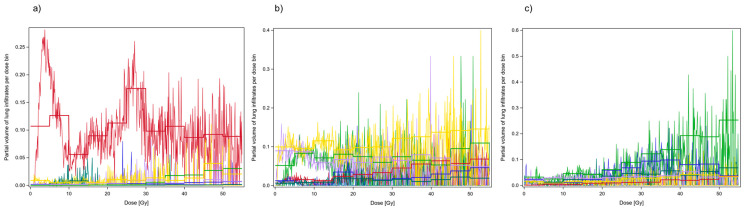
Partial volume of lung infiltrates within different density ranges per 0.1 Gy dose step. (**a**) density stratum from −850 HU to −800 HU; (**b**) density stratum from −650 HU to −600 HU; (**c**) density stratum from −350 HU to −300 HU. Data from different patients are coded in different colors. The 5 Gy stepped regression curves were obtained by the mixed model for the partial volume of lung infiltrates within the respective density range in dependence on dose and density.

The mixed model used to analyze the dose–effect relation for the partial volumes of infiltrates per dose and density bin could adjust for patient-dependent random effects, independently acting on each density and dose bin per patient. Partial lung infiltrate volumes of a given density within different 50 HU density bins are shown in Figure 2 in 0.1 Gy dose steps over the whole dose range. The fitted curves with steps between the 5 Gy dose bins were obtained by the mixed model containing 5 Gy dose bins and the 50 HU density bins as fixed classification effects. Note that considerable statistical fluctuations occurred when separating infiltrate volumes in small dose steps of 0.1 Gy according to their density into 50 HU density bins. Highly significant systematic effects of 5 Gy dose bins and 50 HU density bins as classification variables were detected in the partial volumes of lung infiltrates of the respective density (both *p* < 0.0001, F-test). No significant interaction existed between the dose dependence and the dependence on the density bin (*p* > 0.5, F-test).

**Figure 3:**
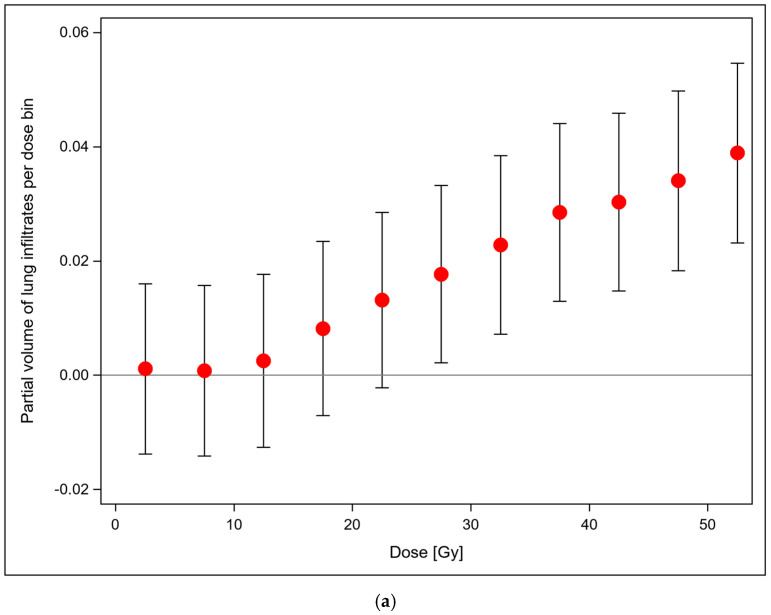

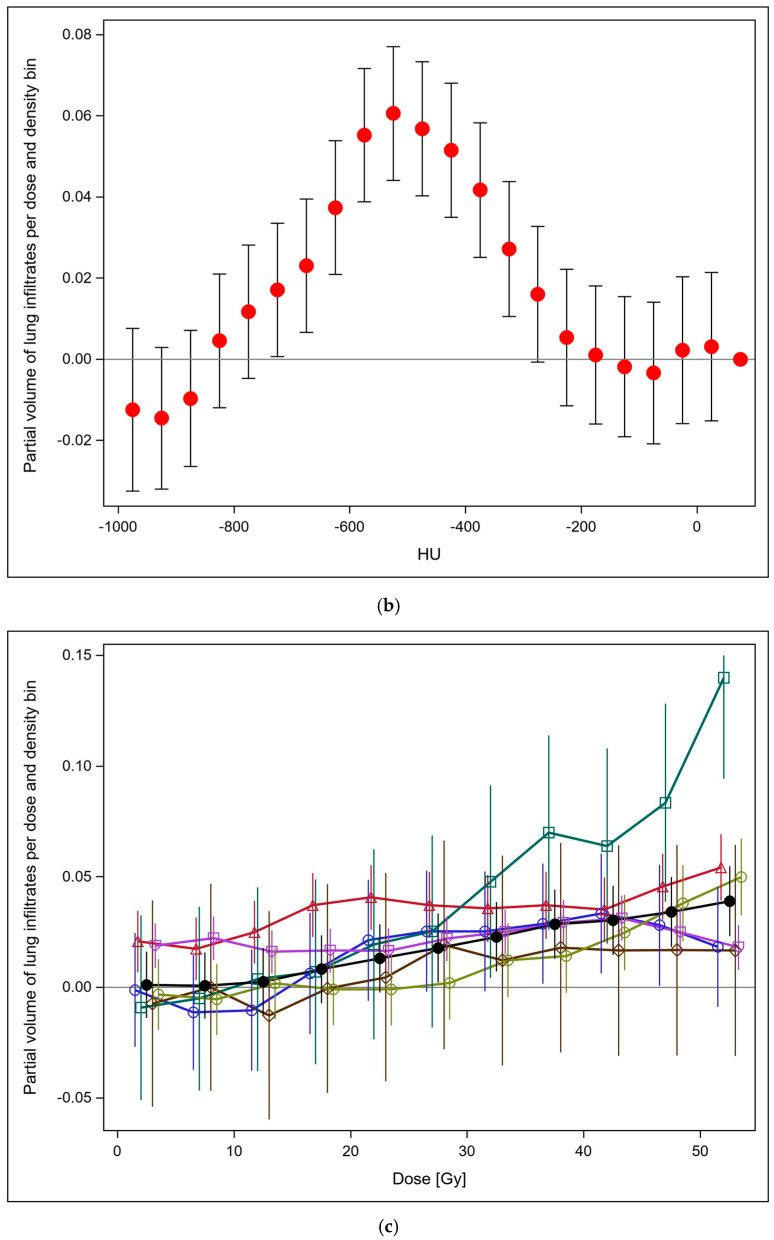
(**a**) Systematic dose-dependence term of partial volumes of lung infiltrates on the 5 Gy dose bins for each 50 HU density stratum from the mixed model (*p* < 0.0001, F-test). There was no significant interaction between the effects of dose and density stratum on the partial infiltrate volumes. (**b**) Systematic dependence of the partial infiltrate volume per dose bin on the different 50 HU density strata of the infiltrates for each 5 Gy dose bin from the mixed model (*p* < 0.0001, F-test). (**c**) Dose–response relationships per patient, significant dose response for each patient with *p*-values < 0.0001 for each patient. Data from different patients are coded in different colours.

The fixed effect of the 5 Gy dose bin on the partial volume of lung infiltrates per dose bin in each density stratum is given in Figure 3a. As a classification effect, no functional form of the dose–effect relation was assumed and a monotonously increasing dose dependence was found, approaching 3% partial infiltrate volume in the (50–55) Gy dose bin. The dose–response relation was not dependent on a single patient. Leaving one patient out in a loop over all patients, the dose response remained monotonously increasing and significant at *p* < 0.0001.

Figure 3b shows the fixed effect of the respective density stratum on the partial volume of lung infiltrates in that density range per 5 Gy dose bin. The relation was found to be unimodal with a maximum infiltrate volume at densities around −500 HU. The location of this peak was modulated by the random effects from patient to patient. As there was no interaction effect between the fixed effects of dose and density, these effects are dose independent. Over all 22 HU density strata, this dose-independent effect amounts to a partial infiltrate volume of 37.3% ± 7.0% in each dose bin.

The analysis of the dose–response relationships per patient (Figure 3c) showed a significant dose response for each patient with *p*-values < 0.0001. Thus, the results do not depend on a single patient.

We analyzed the binned dose–response by a linear quadratic model in addition to the step model, and found a similar performance (AIC = −1,444,140) in both models, with a significant linear but insignificant quadratic term (AIC = −144,137); therefore, a linear dose–response relationship is adequate for grade ≥ 3 pneumonitis cases. The mixed model with a constant dose–response relation had an AUC of −143,994 and was therefore significantly worse as shown by the larger AIC. We also fitted the linear dose–response relations with the mixed model to all patients and found significantly positive slopes for all the patients with all associated *p*-values < 0.002. The largest slope was 0.02407 + 0.000173 proportion of pulmonary infiltrates per density bin per Gy and the smallest was 0.00159 + 0.00049 proportion of pulmonary infiltrates per density bin per Gy (median 0.000696).

**Figure 4:**
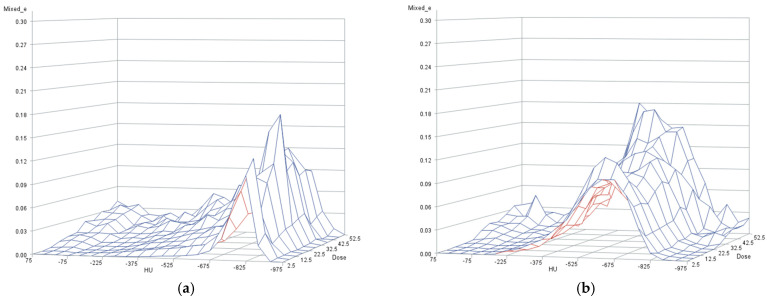

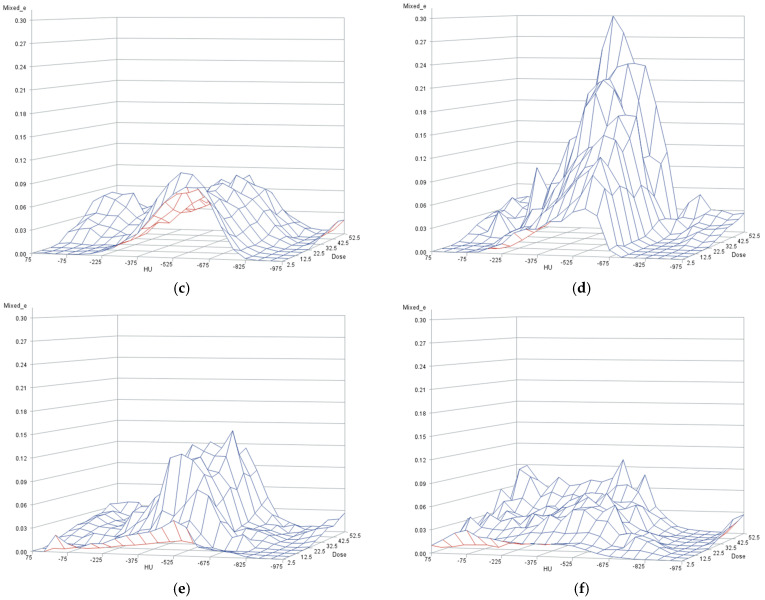
Distribution of the partial volumes of lung infiltrates per 5 Gy dose and 50 HU density bin for the different patients ((**a**–**f**), from upper left to lower right), as estimated by the mixed model. The height represents the partial volume of lung infiltrates per 5 Gy and 50 HU bin, the breadth reflects the volume per HU bin and the depth reflects the volume per dose bin.

The distribution of the partial infiltrate volumes per dose and density bin is shown in Figure 4 for the six patients with high-grade pneumonitis, as obtained from solutions for the fixed and random effect parameters by adapting the mixed model to the 72,600 observed partial infiltrate volumes in 0.1 Gy dose steps over 22 density bins of 50 HU width. Figure 4 shows that the random effects, depending on the patient, the dose and the density bin, modify the distributions of the partial infiltrate volumes from patient to patient. The distributions of the random effects sorted according to the dose or density bin are shown in Figure S1. In addition, we performed the above analysis in normal scores computed from the ranks of the partial volume of infiltrates and again found a monotonously increasing dose–response relation for the partial infiltrate volumes (*p* < 0.0001, F-test). Further research is necessary to explain the biological mechanisms behind these random effects.

**Figure 5:**
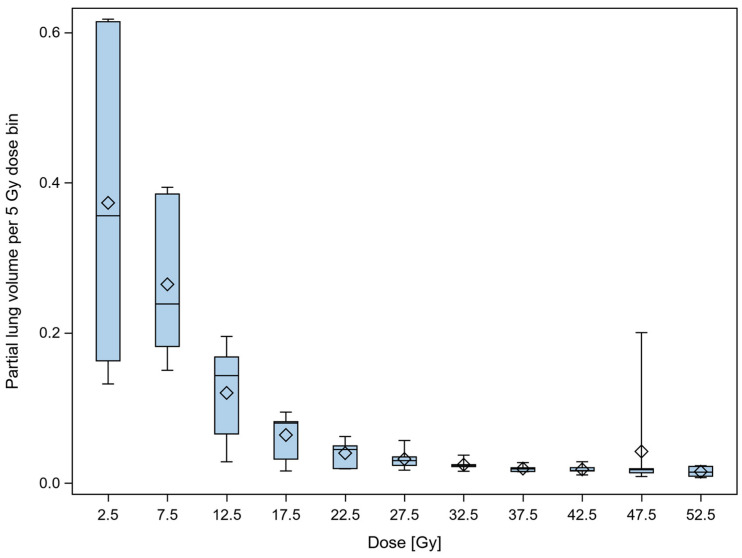
Box plot depicting the distributions of partial lung volumes of the 6 patients with high-grade pneumonitis over the adjacent 5 Gy dose bins. The boxes span the interquartile ranges, the horizontal lines within the boxes indicate the medians, and the diamond marker indicates the means. The whiskers extend from the 10th to the 90th percentile. There were significant differences between dose bins (*p* < 0.0001, Kruskal–Wallis test). Partial volumes per dose bin decreased with increasing dose.

Figure 4 also demonstrates that there are considerable partial volumes of lung infiltrates in the lowest dose bins. The partial volumes of lung infiltrates range in the (0–5) Gy dose bin from 17.9% to 58.4% (median 31.4%), and in the (5–10) Gy bin from 9.1% to 61.2% (median 36.6%). Over all dose bins, the percentage of total infiltrate volumes outside the PTV compared to total lung volumes outside the PTV range from 15.5% to 60.0% from patient to patient (median 39.8%). As the partial lung volumes per 5 Gy dose bin are largest in the low-dose region below 25 Gy (Figure 5), the accumulated absolute infiltrate volumes over all dose bins that are attributable to the systematic dose–response relation, shown in Figure 3a, are limited. They amount to 12.6% to 20.7% of the total infiltrate volume per patient (mean 16.6%). Therefore, a limited, but considerable proportion of the infiltrates follow a systematic dependence on radiation dose, while the majority of the infiltrates are in the low-dose region.

**Figure 6:**
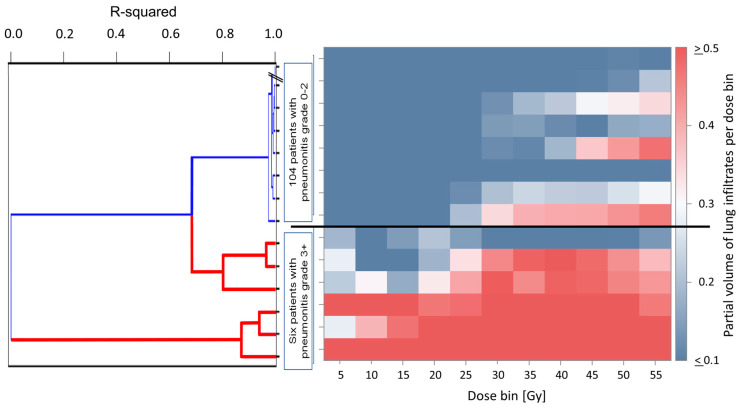
Dendrogram for 110 patients with all pneumonitis grades clustered according to partial volume of lung infiltrates in 5 Gy dose bins from 0 to 55 Gy. There was a good separation between patients with high-grade pneumonitis (grade ≥ 3; red leaves) and those without (blue leaves). For the sake of clarity, only data from the 14 patients in the last agglomerated clusters are shown.

In order to compare the partial volume of infiltrate data from patients with high-grade pneumonitis in this study with the respective data from 104 patients with grade ≤ 2 pneumonitis from our previous study [28], we performed agglomerative hierarchical clustering using the partial infiltrate volumes per 5 Gy dose bins. As the infiltrate volumes were not separated into density strata in the previous analysis, we accumulated the infiltrate volumes per dose bin over all density strata. This unsupervised clustering method showed a very good separation of the six patients with grade ≥ 3 pneumonitis from those with lower-grade pneumonitis according to the dose–response functions for partial lung infiltrate volumes. Figure 5 shows the dendrogram of the last 19 generations of clusters joined together, while the six high-grade patients were agglomerated in the last 6 steps, indicating the large separation from the other patients. The dendrogram shows the six patients with grade ≥ 3 pneumonitis (red leaves) well separated from those with grade ≤ 2 pneumonitis (blue leaves). The heat map in Figure 6 shows the distribution of the partial infiltrate volumes per 5 Gy dose bin of the patients sorted according to the dendrogram into the different rows. Receiver operator characteristic analysis by a logistic model showed a very good separation between patients with high-grade pneumonitis and those with grade ≤ 2 pneumonitis by the partial volumes of lung infiltrates in the [0–5) Gy dose, characterized by an area under the curve (AUC) value of 1.0 (*p* < 0.0001, likelihood-ratio test). The same was observed in each of the dose bins up to 20 Gy. The patients with grade 4 and 5 pneumonitis had the highest partial volumes in the low-dose region in the lowest three dose bins. At higher dose bins, the AUC values decreased below 1.0 but stayed above 0.95 for all dose bins with lower 95% confidence interval limits for AUC above 0.90 (*p* < 0.0001, chi^2^ test) (confidence intervals for dose bins are available in the Supplementary Materials). Comparing the partial volumes of lung infiltrates in the [0–5) Gy dose of patients with pneumonitis grade 2 alone with those of high-grade pneumonitis, the same AUC values of 1.0 were obtained (*p* < 0.0001, likelihood ratio test).

## 4. Discussion

Liu et al. conducted a meta-analysis of 248 studies including over 28,000 patients with locally advanced non-small cell lung cancer (NSCLC) treated with definitive radiochemotherapy without immunotherapy [46]. They found that the average rate of severe (grade ≥ 3) pneumonitis was 4.6% (95% CI: 3.4–5.9%) in non-Asian patients and slightly higher at 6.5% (95% CI: 5.6–7.1%) in Asian patients [46]. The grade 5 pneumonitis rate in non-Asian versus Asian patients was found to be 0.1% (95% CI: 0.0–0.2%) versus 0.6% (95% CI: 0.3–0.9%). This rate was, at least in non-Asian patients, somewhat lower than after radiochemotherapy with immunotherapy consolidation. For comparison, the pooled median incidence of grade 5 pneumonitis after radiochemotherapy and immunotherapy consolidation in the meta-analysis by Yang was 0.8% (95% CI: 0.3–1.2%) [47]. Meta-analyses revealed a pooled mean incidence of grade ≥ 3 pneumonitis between 5.7% and 8% (95% CI: 3.0–8.5%) [2,3,4,5,47]. The proportion of patients with grade ≥ 3 pneumonitis in this range was 4.5%. The proportion of patients who developed a grade ≥ 3 pneumonitis after immunotherapy or chemoimmunotherapy alone for advanced NSCLC was slightly lower in the meta-analysis by Saowapa et al. 2024, with a pooled incidence of 2.0 (95% CI: 1.6–2.4%), although there were slight differences between PD-1 and PD-L1 inhibitors [48]. Similar pooled incidences were found in the meta-analysis of Kong et al. 2023 and Ding et al. [49,50]. The risk of grade ≥ 3 pneumonitis attributable to PD-1 or PDL1 antibodies alone or in combination with chemotherapy regimens was 0.76% (95% CI: 0.38–1.34) in randomized trials for triple-negative breast cancers [51], 1.0% (95% CI: 0.7–1.4%) in urologic cancer patients [52], and 0.8% in melanoma [53]; therefore, it was lower than in lung cancer.

The radiologic pattern of infiltrates in checkpoint inhibitor-induced pneumonitis on a chest CT can be classified into organizing pneumonitis, nonspecific interstitial pneumonitis, hypersensitivity pneumonitis, acute interstitial pneumonitis—acute respiratory distress syndrome and bronchiolitis. The organizing pneumonitis pattern is the most common [54,55,56,57]. All of them are most often bilateral. The pattern of organizing pneumonitis often includes patchy infiltrates, predominantly in the middle or lower regions of the lungs, while NSIP infiltrates are predominantly located in the base and subpleural regions and are accompanied by ground-glass opacities and reticular opacities. A hypersensitivity pattern includes diffuse or centrilobular ground-glass opacities, predominantly in the middle or upper lobes of the lungs.

Machine learning algorithms utilizing features extracted from manually segmented lung infiltrates were assessed without incorporating radiation dose distribution data [35,36,58,59,60]. These models aimed to differentiate between pneumonitis induced by radiation therapy and that caused by immune checkpoint inhibitors. The classifiers demonstrated good performance, achieving area under the curve (AUC) values ranging from 0.76 to 0.94 [35,36,58,59,60]. However, some models performed less well in patients who were previously exposed to both radiotherapy and checkpoint inhibitors with an AUC of 0.54 [58]. Wu et al. discussed in a review how the quantitative feature extraction from medical images can improve early lung cancer detection and clinical decision-making. Applications in differentiating benign from malignant pulmonary nodules, assessing tumor invasiveness, predicting prognosis, and supporting personalized treatment strategies may enhance diagnosis and enable advanced imaging analytics to be translated into practical clinical tools for lung cancer screening and management [61]. Integrated nomograms that combine semantic CT features with radiomic features to predict invasive pulmonary adenocarcinoma in patients with persistent subsolid nodules demonstrated high diagnostic accuracy and good validation performance [62]. The value of nomograms lies in providing a clinically applicable, noninvasive predictive tool that may improve pretreatment risk stratification and guide more precise management of patients.

Comparing the infiltrate pattern of checkpoint induced pneumonitis with that of radiation-induced pneumonitis, Chen et al. found that checkpoint inhibitor pneumonitis was more often bilateral, involved more lobes and was less likely to have sharp borders [63]. The severity of pneumonitis increases with the spatial extent of pulmonary infiltrates. Bilateral and multifocal changes were more often seen in grade ≥ 3 pneumonitis than in grade ≤ 2 pneumonitis [63]. Diffuse distribution of infiltrates, AIP/ARDS pattern of infiltrates, and the proportion of infiltrates in all lung zones, especially a proportion of more than 5% in diffuse infiltrates, were associated with severe pneumonitis in the study by Zhang et al. [64].

Several studies found infiltrates in the low-dose region in patients with clinical pneumonitis after radiochemotherapy and immunotherapy [34,63,65,66]. Smesseim et al. defined infiltrates outside the radiation fields as a hallmark of checkpoint inhibitor induced pneumonitis after combined radiation therapy and durvalumab consolidation therapy [6]. Similarly, the ESTRO clinical practice guideline for the diagnosis and treatment of radiation-induced pneumonitis states that radiological changes that are strictly limited to the irradiated area strongly support the diagnosis of radiation pneumonitis [6,67]. In this study, the proportion of infiltrates in the low-dose region below 5 Gy was the most significant factor that could differentiate between grade ≤ 2 and grade > 2 pneumonitis after radiochemotherapy and immunotherapy consolidation with an AUC of 0.9.

In the present study, we analyzed the dose dependency of the proportion of infiltrates in patients with grade ≥ 3 pneumonitis. Importantly, we adjusted for patient-dependent random effects on the proportion of infiltrates, which can vary independently from dose range to dose range and from density stratum to density stratum. A monotonically increasing dose–response relationship was observed, accounting for a mean of 16.6% of the total pneumonitis infiltrate volume per patient. This finding supports the notion that pneumonitis following combined radiochemotherapy and durvalumab treatment arises from the interplay of both modalities, rather than being attributable solely to radiotherapy or immune checkpoint inhibition. This finding distinguishes it from monofactorial pneumonitis, which is triggered by one of the two treatments.

Preclinical studies in mice have demonstrated that the combination of radiation therapy and checkpoint inhibitor immunotherapy can exacerbate lung toxicity [68,69]. Notably, the severity of these side effects depends on the sequence in which the two therapies are administered [68,69]. Several studies analyzed the risk of pneumonitis following radiochemotherapy with subsequent immunotherapy consolidation, focusing on the absolute lung volume receiving more than 5 Gy, 20 Gy, or 40 Gy. Associations were observed between pneumonitis risk and some of these radiation dose thresholds [70,71,72,73,74]. Others did not find such an association [75]. In the present study, we found no significant differences in total lung radiation exposure (V5 or V20) between patients who developed up to grade 2 pneumonitis and those who developed high-grade pneumonitis following radiochemotherapy and immunotherapy. This suggests that interindividual variability in sensitivity to the combined treatment plays a major role. Instead, the analysis performed in the present study compared the intra-patient proportion of infiltrates of the same density in the high- and low-dose regions of the lung, adjusting for patient-dependent dose-independent random factors in each dose and density bin. The partial volume of lung infiltrates as well as the mean lung density was correlated with the severity of symptoms in viral pneumonia. Good correlations with disease severity were found for partial volumes of higher-density infiltrates with HU values using cut-off values between −800 and >−600 HU with larger lung arteries excluded [76,77,78]. Severity of symptoms also correlated with the mean lung density using unenhanced CT scans. However, using contrast-enhanced pulmonary CT, density differences between healthy lungs and lungs with atypical pneumonia are diminished [78]. In the present study, CT pulmonary angiograms alone were available at peak pneumonitis. Therefore, we did not correlate density distributions with disease severity. Instead, we adjusted for random density changes from patient to patient in each dose and density bin in order to detect intra-patient dose–effect relations.

The most important factor, associated with grade ≥ 3 pneumonitis in comparison to grade ≤ 2 pneumonitis during follow-up, was the percentage of infiltrates in the (0–5) Gy bin. All patients with high-grade pneumonitis had percentages of infiltrates > 15% in that dose bin. As such percentages of infiltrates in the low-dose region indicate severe forms of pneumonitis, intensive immunosuppressive treatment is indicated. As infiltrates after definitive radiochemotherapy and durvalumab consolidation were found to have radiation dose-dependent and also independent components, a mixed picture of checkpoint inhibitor and radiation-induced pneumonitis emerges. From a mechanistic perspective, dose-independent infiltrates suggest the involvement of molecular pathways that are not strictly governed by local dose distribution. By disrupting immune checkpoint pathways that maintain self-tolerance, immune checkpoint inhibitors (ICIs) can provoke immune-related adverse events [79,80]. However, precise mechanisms underlying lung injury in immune checkpoint pneumonitis are not well understood and are the subject of ongoing research [81,82]. A dysregulation of CD4+ T-cells, central memory T-cells, T-helper 17.1 cells and proinflammatory macrophages and the upregulation of specific metabolites such as autoantibodies seem to be major drivers of immune-related inflammation [81]. The mechanisms underlying checkpoint inhibitor–induced pneumonitis in lung cancer patients without radiotherapy involve T-cell expansion. This expansion predominantly affects interferon-γ–producing central memory T cells. These cells accumulate in lung tissue at sites of pulmonary infiltrates in response to immunotherapy [80,81,82]. Activated T cells present in the tumor micromilieu may relocate to normal lung compartments and are activated by cross-reactive antigens due to checkpoint inhibitor therapy. In addition, an upregulation of a humoral response to immunotherapy, elevated proinflammatory cytokines and upregulated chemokine receptors were observed in lung tissue of patients with checkpoint inhibitor-induced pneumonitis [80,81,82].

Checkpoint inhibitor–associated pneumonitis is more frequently steroid-resistant than radiation-induced pneumonitis [83,84]. In addition, it may deteriorate within days [85], underscoring the need for a timely escalation of immunosuppressive therapy [86,87]. Consequently, lung voxels exposed to higher doses might be assigned a higher weight in the risk term of the objective function for treatment plan optimization.

The limits of the study lie in the limited number of patients seen in a single high-volume center. The results of the study may be heavily dependent on specific patient characteristics. In addition, as pneumonitis is a dynamic process, the captured CT studies do not necessarily capture the same phase of the pneumonitis course. Furthermore, the accuracy of the analysis also depends on the deformation of the planning CT on the follow-up CT with infiltrates, which sometimes show greatly altered densities in patients with severe pneumonitis. However, the hybrid deformation used in the present study was supervised for every single patient by two expert radiation oncologists and radiologists.

## 5. Conclusions

High-grade pneumonitis following radiochemotherapy and durvalumab consolidation in patients with locally advanced NSCLC is associated with infiltrate volumes exceeding 15% of the lung in regions receiving less than 5 Gy. A radiation dose-dependent component accounts for a median of 24% of the total infiltrate volume per patient. These results indicate that pneumonitis involves contributions from both radiochemotherapy-induced and immunotherapy-related mechanisms.

## Figures and Tables

**Table 1:** Baseline characteristics of study population with a grade ≥ 3 pneumonitis and dosimetric parameters.

Age [Years], Median (Range)	73.5 (66.0–81.0)
Sex, male/female	3/3
Karnofsky index [%], median (range)	80 (70–80)
Time from start of immunotherapy until infiltrates-CT [months], median (range)	3.5 (2–14)
Total dose [Gy], median (range) Fractionation scheme	60 (54–66) 5 × 2 Gy/w
Mean Lung dose [Gy], median (range)	16.8 (13.9–18.4)
Lung V5, [%], median (range)	67.0 (45.3–92.2)
Lung V20, [%], median (range)	27.7 (22.9–31.2)
Mean HU of pulmonary infiltrates, median (range)	−506 (−751.9–−316.8)
Pneumonitis grade 3/4/5	4/1/1

## Data Availability

Data are available from the corresponding author to researchers upon reasonable request.

## Supplementary Materials

The following supporting information can be downloaded at: https://www.mdpi.com/article/10.3390/diagnostics16060827/s1, Figure S1: Distribution of the random effects on partial infiltrate volumes of respective density per dose and density bin, sorted according to adjacent 5 Gy dose bins. The mean is indicated by a diamond, and the boxes extend from the first to the third quartile. The median is indicated by a horizontal line within the box. The whiskers extend from the 10th to the 90th percentile, Figure S2. Distribution of the random effects on partial infiltrate volumes of indicated density over the different 5 Gy dose bins for different patients, sorted according to adjacent 50 HU density bin. The mean of the distribution is indicated by a diamond, and the boxes extend from the first to the third quartile. The median is indicated by a horizontal line within the box. The whiskers extend from the 10th to 90th percentile.
