# Supplementary material for: Radiation Dose-Dependent and -Independent Pulmonary Infiltrates in Patients with High-Grade Pneumonitis After Radiochemotherapy and Durvalumab Consolidation for Stage III NSCLC
Source: Diagnostics (Basel). 2026 Mar 11;16(6):827. doi: 10.3390/diagnostics16060827 (PMC13025436; doi:10.3390/diagnostics16060827)
Supplement: Supplementary file 1 [file diagnostics-16-00827-s001.zip › diagnostics-4066708-supplementary.pdf]

Supplement Figure S1.

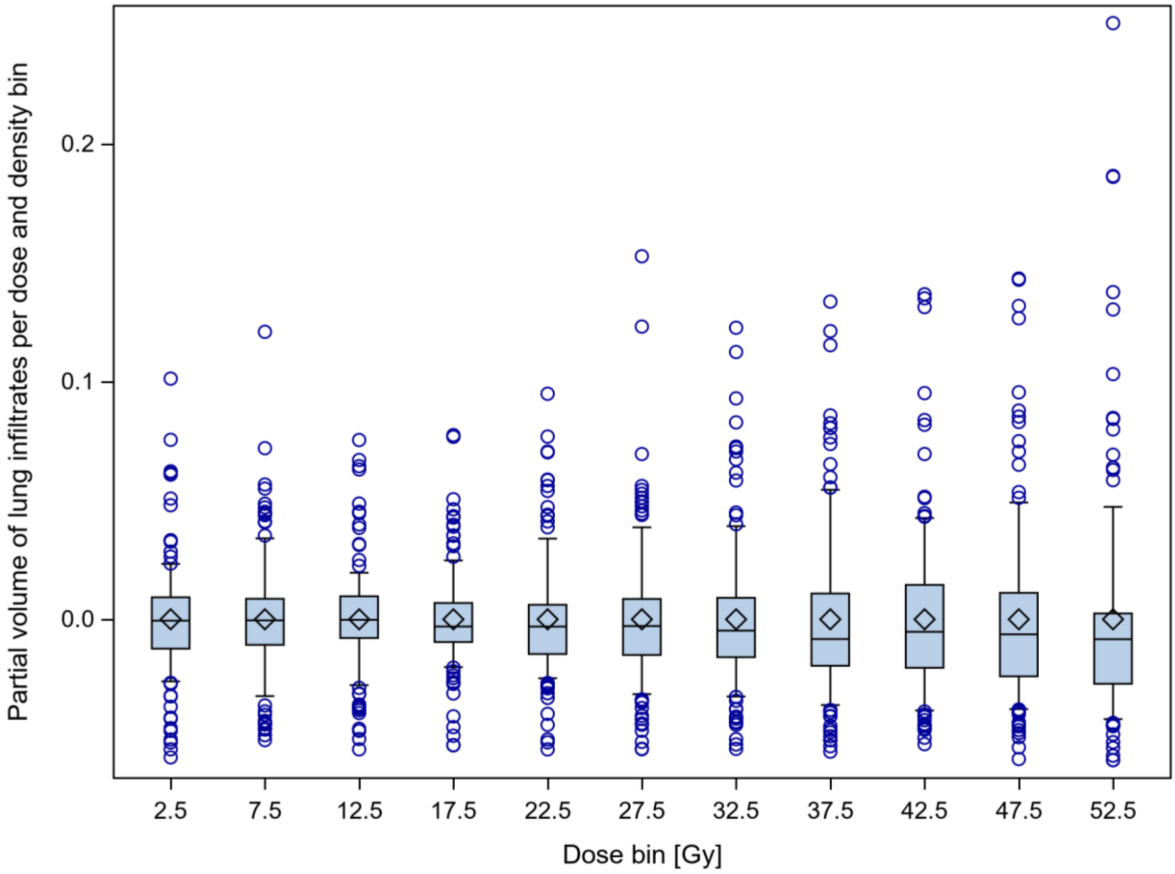

Suppl. Figure S1. Distribution of the random effects on partial infiltrate volumes of respective density per dose and density bin, sorted according to adjacent 5 Gy dose bins. The mean is indicated by a diamond and the boxes extend from the first to the third quartile. The median is indicated by a horizontal line within the box. The whiskers extend from the 10th to 90th percentile.

Supplement Figure S2.

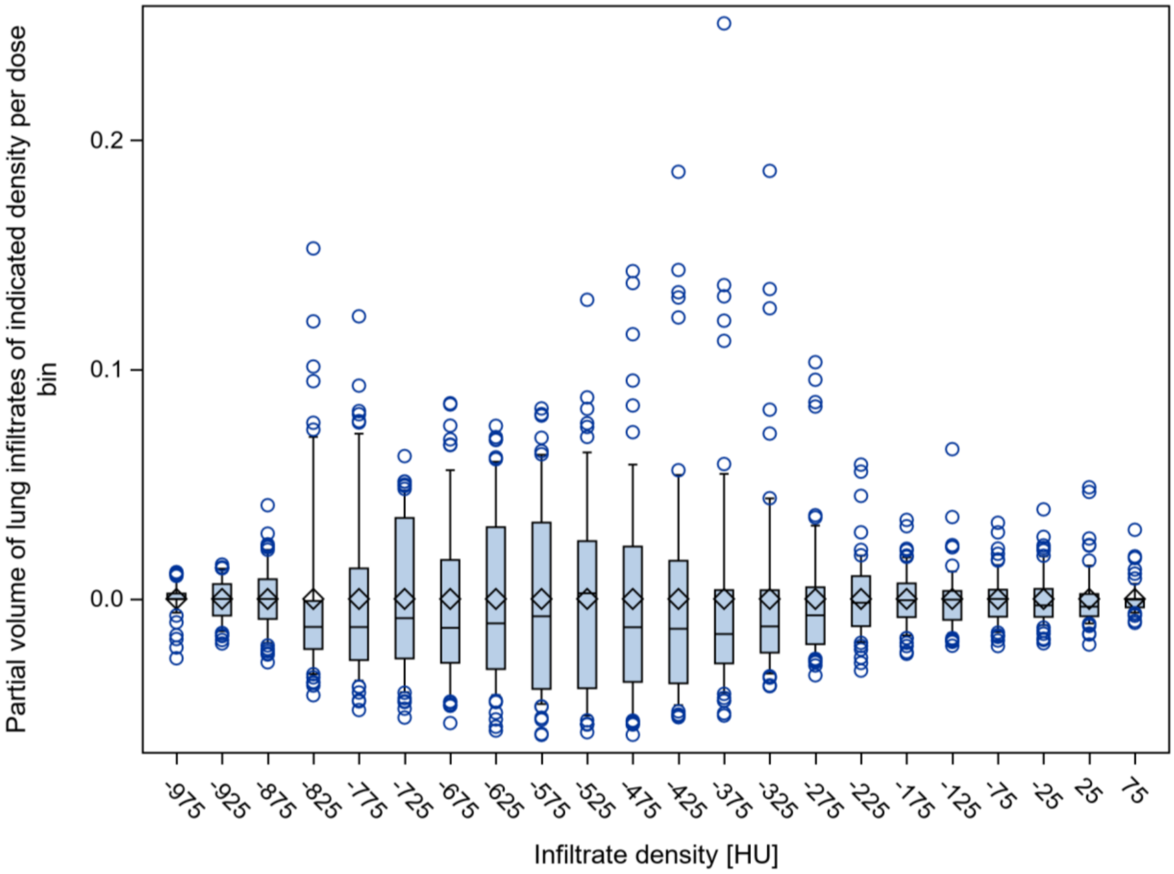

Suppl. Figure S2. Distribution of the random effects on partial infiltrate volumes of indicated density over the different 5 Gy dose bins from the different patients sorted according to adjacent 50 HU density bin. The mean of the distribution is indicated by a diamond, the boxes extend from the first to the third quartile. The median is indicated by a horizontal line within the box. The whiskers extend from the 10th to 90th percentile.

Confidence intervals for the AUC values for the separation with or without high grade pneumonitis by the partial volumes of infiltrates per dose bin using logistic regression

AUC for the [0-5) Gy dose bin: 1.000 [95% CI: 0.9991 – 1.000]

AUC for the [5-10) Gy dose bin: 1.000 [95% CI: 0.9991 – 1.000]

AUC for the [10-15) Gy dose-bin: 1.000 [95% CI: 0.9991 – 1.000]

AUC for the [15-20) Gy dose-bin: 1.000 [95% CI: 0.9991 – 1.000]

AUC for the [20-25) Gy dose-bin: 0.9984 [95% CI: 0.9940 – 1.000]

AUC for the [25-30) Gy dose-bin: 0.9872 [95% CI: 0.9606 – 1.000]

AUC for the [30-35) Gy dose-bin: 0.9792 [95% CI: 0.9583 – 1.000]

AUC for the [35-40) Gy dose-bin: 0.9696 [95% CI: 0.9086 – 1.000]

AUC for the [40-45) Gy dose-bin: 0.9712 [95% CI: 0.9133 – 1.000]

AUC for the [45-50) Gy dose-bin: 0.9663 [95% CI: 0.9991 – 1.000]

AUC for the [50-55) Gy dose-bin: 0.9583 [95% CI: 0.9013 – 1.000]

AUC values were considered as significant at alpha <0.001 at p-values for each dose bin met this criterion
